# Sensory and cultural acceptability tradeoffs with nutritional content of biofortified orange-fleshed sweetpotato varieties among households with children in Malawi

**DOI:** 10.1371/journal.pone.0204754

**Published:** 2018-10-18

**Authors:** Marijke Hummel, Elise F. Talsma, Ati Van der Honing, Arthur Chibwana Gama, Daniel Van Vugt, Inge D. Brouwer, Charles Spillane

**Affiliations:** 1 Plant & AgriBiosciences Research Centre (PABC), Ryan Institute, National University of Ireland Galway, Galway, Ireland; 2 International Potato Center (CIP), Lilongwe, Malawi; 3 Division of Human Nutrition, Wageningen University, Wageningen, The Netherlands; 4 Harvest Plus, International Center for Tropical Agriculture (CIAT), Cali, Colombia; Indiana University Bloomington, UNITED STATES

## Abstract

**Background:**

Biofortified orange-fleshed sweetpotato (OFSP) varieties are being promoted to reduce vitamin A deficiencies due to their higher beta-carotene content. For OFSP varieties to have impact they need to be accepted and consumed at scale amongst populations suffering from vitamin A deficiencies.

**Objective:**

We investigated the sensory and cultural acceptability of OFSP varieties amongst households with children aged between 2–5 years old in two areas in Central and Southern Malawi using an integrated model of the Theory of Planned Behavior (TPB) and the Health Belief Model (HBM).

**Methods:**

Sensory acceptability was measured using a triangle, preference and acceptance test using three OFSP varieties and one control variety, among 270 adults and 60 children. Based on a food ethnographic study, a questionnaire on cultural acceptability was developed and administered to 302 caretakers. Data were analyzed by calculating Spearman’s correlations between constructs and multiple linear regression modeling.

**Results:**

The sensory evaluation indicates that all three OFSP varieties are accepted (scores >3 on 5-point scale), but there is a preference for the control variety over the three OFSP varieties. Almost all caretakers are intending to frequently prepare OFSP for their child in future (97%). Based on regression analysis, the constructs ‘subjective norms’ (β = 0.25, p = 0.00) reflecting social pressure, and ‘attitudes toward behavior’ (β = 0.14 p = 0.01), reflecting the feelings towards serving their child OFSP, were the best predictors for caretakers’ behavior to prepare OFSP for their child.

**Conclusions:**

Our study shows that both sensory and cultural attributes can influence acceptability of varieties and consumption amongst households with children. Considering these attributes can improve the impact of biofortified crops in future programming, by reducing Vitamin A deficiencies through the intake of these nutrient-rich crops.

## Introduction

Sweetpotato (*Ipomoea batatas (L*.*) Lam*) is one of world’s most important crops for food and nutrition security, particularly in Sub-Saharan Africa, parts of Asia, and the Pacific Islands [1, 2]. Malawi is the main producer of sweetpotatoes in Sub-Saharan Africa, with an average production of 3.9 million ton per year in the period 2012–2014 [2]. From a nutrition perspective, sweetpotato roots are a good source of carbohydrates, fiber and vitamins B, C and E [3]. Most of the varieties of sweetpotato currently grown and consumed in Sub-Saharan Africa are white- or yellow-fleshed, and contain little beta-carotene [2]. In recent years, breeding programs have developed improved biofortified orange-fleshed sweetpotato (OFSP) varieties that are a good source of beta-carotene, a precursor of vitamin A [4].

Vitamin A deficiency is one of the major nutritional deficiencies in the world, affecting 190 million preschool children globally [5]. Micronutrient surveys conducted in Malawi in 2001 and 2009 reported that 59% and 23% of preschool children were vitamin A deficient [6]. Recent data on vitamin A deficiency however suggests that only 4% of preschool children living in rural areas in Malawi are vitamin A deficient [7], which is defined by the World Health Organization as a mild public health problem [8]. Possible explanations for this drop in deficiency rates could be the mandatory vitamin A fortification of oil and sugar in Malawi since 2015. Only 67% of preschool children received a vitamin A capsule in the last 6 months [9], hence there remains a need for a more sustainable and cost effective approach to reduce vitamin A nutrition deficiencies.

Biofortification strategies to improve human nutrition can be complementary to supplementation, dietary diversification, and fortification initiatives to combat vitamin A deficiency. Biofortification is a food-based approach to combat micronutrient malnutrition through breeding staple crops that have higher levels of micronutrients (e.g., iron, zinc, beta-carotene). Biofortification has been shown to be effective to alleviate micronutrient deficiencies in several populations [10]. The HarvestPlus Program has met pre-set breeding goals for OFSP with a beta-carotene level of 3200 ug/100g OFSP, to meet the daily requirements for vitamin A when consuming 100 grams of OFSP per day for a child aged 4–6 years [10]. The consumed beta-carotene is converted in the human body to vitamin A, which is one of the essential micronutrients for human nutrition [11]. Hence, OFSP consumption has major potential to contribute to decreases in vitamin A deficiency rates in children as well as adults, which has been shown by both efficacy and effectiveness studies [4, 12].

The first OFSP variety Zondeni was locally available in farmers’ fields and officially recognized in Malawi in 2008, followed by an additional five varieties that were released in 2011 through a breeding program [13]. The OFSP varieties have different visual phenotypes and taste than the pre-existing varieties of sweetpotato used by farmers. The orange color intensity of OFSP varieties is associated with higher beta-carotene levels and lower dry matter content [14, 15]. Such trait changes can influence sensory and cultural acceptability, where newly introduced varieties have to remain acceptable to consumers if they were to have the intended effect of improving the vitamin A status of the target consumers. As acceptability can differ due to cultural and demographic factors, it is important to conduct research on each country-crop combination [16]. Talsma *et al*. have reviewed nine studies on the sociocultural drivers and determinants of acceptance and adoption of OFSP [17]. Overall, these studies indicated that acceptability and adoption of OFSP were high in areas where it was promoted. While OFSP has been promoted throughout Malawi since 2009, no in-depth research has been published on identifying factors that can influence the acceptability of consuming OFSP. To assess such cultural acceptability an integrated model, combining the Theory of Planned Behavior (TPB) and the Health Belief Model (HBM), can be used to investigate food or health-related behavior [18].

The TPB model assumes that the intention to perform a behavior, in our case consumption of OFSP, is closely related to the behavior itself. The intention to perform this behavior can be predicted by attitudes toward the behavior, subjective norms and perceived behavioral control [19]. The HBM is used for explaining and predicting acceptance of health-related recommendations. It combines individual perceptions and modifying factors to a likelihood of action, eg. of adopting a certain behavior, in our case OFSP consumption. The most important elements are the perceived susceptibility and threat of the health problem, the cues to action to adopt the behavior and the perceived benefits of the preventive action [20]. This combined TPB/HBM model has been used to investigate the acceptance of foods such as amaranth, iron fortified soya sauce, fonio and yellow cassava, [18, 21–23], but has to date not been used to investigate the acceptability of OFSP.

The aim of our study was to investigate the sensory and cultural acceptability of OFSP amongst households with children between 2–5 years old in two areas in Central and Southern Malawi using the integrated model of the TPB and HBM.

## Material and methods

### Ethics statement

Written informed consents were collected among research participants or caretakers before the start of the study and all children were asked for their verbal consent. Ethical clearance for this research project was obtained from the National University of Ireland Galway Research Ethics Committee (Reference 16/FEB/07) and the National Commission of Science and Technology in Lilongwe, Malawi (Protocol number P.06/16/114.).

### Study area

The research was conducted in Central and Southern Malawi in, respectively, the Mngwangwa location in Lilongwe district and Katuli location in Mangochi district. These rural research sites were selected based on their high production levels of sweetpotato and beans, the difference in culture (Chewa ethnic group in Mngwangwa and Yao ethnic group in Katuli) and the presence of collaborating organizations (International Potato Center and Concern Worldwide).

Mngwangwa is situated relatively close to the capital of Malawi, Lilongwe, at a distance of approximately 30 km. Katuli lies more isolated behind hills on 60 kilometers from Mangochi, and is bordering with Mozambique. Malawi has one rainy season stretching from December to April, followed by a long dry season [24]. Both locations can be described as rural areas where over 90% of the population is engaged in agriculture activities [25]. Major crops grown in both locations include maize and groundnuts. In Lilongwe, tobacco, beans and soy are also important crops. Diets are mainly cereal based, in which over 50% of calorie intake is from maize, with Nsima (maize flour mixed with water) as a staple, supplemented with starchy roots (cassava, potatoes), vegetables and beans [26]. Literacy is higher in Lilongwe (64.5%) than Mangochi (57.2%) [25].

### Study participants & sampling

This study consisted of two parts, the sensory evaluation and the cultural acceptability survey. To prevent bias, the two parts of the study were conducted in different areas within the locations. For the sensory study, five villages were identified in each location through random sampling where adults, preschool children aged 4–5 years and their caretakers were interviewed using the method of convenience sampling and central location testing [27]. Sample sizes were large enough to perform analyses stratified by location, except for the preference test for children, where all children were analyzed as one group.

For the cultural acceptability study, participants were selected using multistage sampling, by selecting five areas within the location (each location was subdivided in 20 areas) and then randomly selecting three villages in each selected area. Inclusion criteria for the cultural acceptability study were the presence in the household of a child between 2–5 years of age whom they were taking care of and previous exposure to the OFSP varieties. Therefore, villages were included if there had been any previous OFSP related activity (nutrition promotion, agricultural training, demonstration plots). The list of villages was composed and crosschecked by Agriculture Extension Development Coordinators and field staff from the International Potato Center of the respective areas who were responsible for implementing the OFSP related activities. Probability proportional to (population) size sampling was used to calculate the number of participants per village, in which the sample size for a sub-population (area/village) is weighted proportional to its size (evenly distributed over the 2 locations). Per location 15 villages were included. Participants within each village were randomly sampled using household lists. If the participant selected was not available a new randomly selected household was invited for the interview.

The study was conducted in the period between September and November 2016 by four trained enumerators for the sensory evaluation and five trained enumerators for the cultural acceptability study. Interviews took place in a communal place and were conducted in Chichewa or Yao depending on the preference of the participant. All questionnaires were developed in English and translated to Chichewa and Yao local languages. Correctness of translation was checked by back translation to English. Pretesting was done for both studies and resulted in small changes in explanation to the participants and language used. To assess whether study participants understood the modified 5 point Likert-scale with both faces and checks (i.e. √ symbols) that was used for both parts of the study, an example question was asked which was not related to the research. Caretakers decided about which child the interview was with, when there was more than 1 child eligible; this decision was based on availability of the child to attend the interview. Reasons for exclusion were if the participant did not understand the scale or if the mother could not present a proof of birth date for the child.

### Study measurements

#### Sensory evaluation

For the sensory evaluation one control yellow-fleshed sweetpotato variety (Kenya) and three OFSP varieties (Kadyaubwerere, Chipika and Zondeni) were peeled and cut into roughly equal sized portions between 25 and 40 grams. All varieties were harvested in the dry season from the same irrigated farmer’s field at the Chiwamba location in Lilongwe district, and stored for the same number of days before the tasting took place.

The Kenya variety is a yellow sweetpotato that is widely available in Malawi, and therefore the control variety. Kadyaubwerere is a high-yielding OFSP variety, variety Chipika is a more drought-resistant OFSP, whereas Zondeni is the oldest OFSP variety available in Malawi, and has lower yields [28]. These were boiled until the texture, assessed by a fork by the researchers, was considered right for consumption [29]. To assess sensory acceptability three different tests were done. All participants did a preference test (n = 270), followed by either a triangle (n = 66) or an acceptance test (n = 210).

A triangle test (n = 66) was conducted with the control variety and Kadyaubwerere OFSP variety to determine whether blindfolded participants could perceive a difference in the taste between two sweetpotato varieties. Previous studies indicate that 24–30 participants for a difference test are sufficient to determine a statistical significance for noticeable differences in sensory testing [30]. Three samples of sweetpotato were presented to the blindfolded participant, who was asked to taste and identify the odd sample [27, 30]. A preference test was conducted with adult participants (n = 270) and with 60 children of the age 4–5 years to study the preference between the control variety and the Kadyaubwerere OFSP variety. Sample sizes over n = 20 are possible to analyze, but the ideal sample size is >100 participants when the binomial distribution is very equal to the normal distribution [27]. Participants were given two samples and they indicated whether they preferred the OFSP or the control variety and for what reasons. The same procedure was used for children between 4–5 years [31]. An acceptance test (n = 210) was conducted to evaluate the overall liking of sweetpotato, as well as the liking of the following attributes; taste, colour, smell, texture, starchiness and sweetness. Four sweetpotato varieties were presented to the participants, one control yellow variety of sweetpotato (Kenya) and the three OFSP varieties, Kadyaubwerere, Chipika and Zondeni. The participants were then asked to rate samples for each attribute on a 5-point modified Likert-scale with both smiley faces and checks. All test samples were color-coded and presented in random order. Participants were allowed to swallow the samples and were asked to rinse their mouth with water before the test and after tasting each sample.

#### Cultural acceptability

For the cultural acceptability survey, questionnaire-based interviews were conducted. The questionnaires consisted of two parts. In part one, information on socio-demographics were gathered, followed in part two with statements according to the 13 constructs of the TPB and HBM. These statements were identified based on literature study and a food ethnographic study, consisting of focus group discussions, a food attribute and food difference study, key informant interviews and a pile sorting session [32]. In total, 109 statements were categorized into thirteen constructs as described by Sun *et al*. and Ajzen *et al*. [18, 33]. Anticipated affect was added as an construct to this model for which it was shown that it can explain additional variance in predicting the intention to perform the behavior [33]. The construct ‘attitude towards behavior’ consisted of a maximum of twenty-two questions, whereas there was only one question for the construct ‘health behavior identity’. Respondents were asked to respond to all statements on a 5-point modified Likert scale with both faces and checks to indicate their response ranging from “I completely disagree” to “I completely agree”.

**Fig 1:**
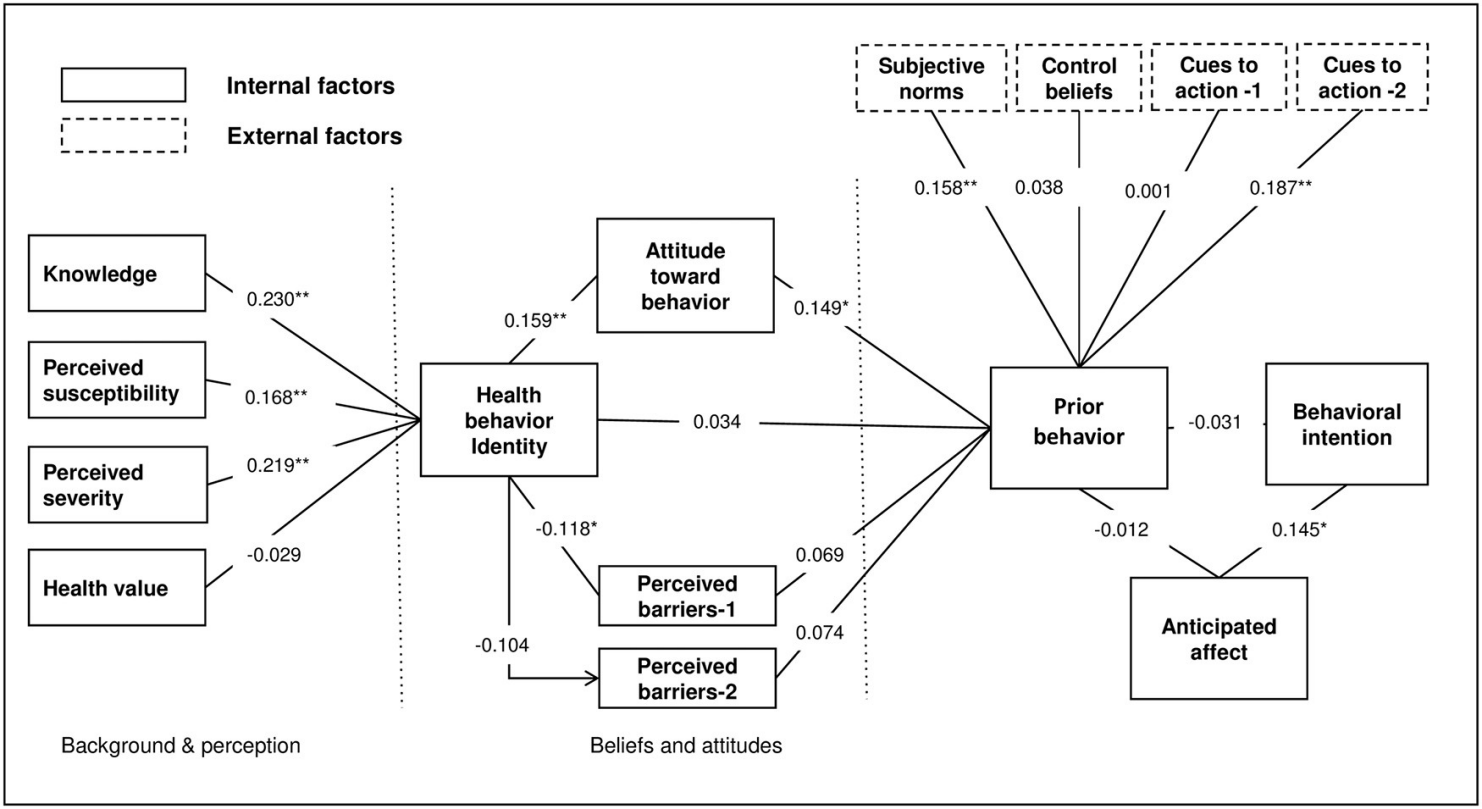
Adjusted combined model of the TPB and the HBM with correlations between the various constructs. (Based on Sun *et al*. 2006) About the model: the model is predicting behavior based on the construct ‘prior behavior’ (frequency of serving the child OFSP in the past when available). Prior behavior is linked to the ‘behavioral intention’ (intention to serve the child OFSP in future), which was the original predictor in the model of Sun et al. Both constructs can be influenced by the anticipated affect (feelings of regret when not serving the child OFSP). All other constructs are divided in three categories. ‘Background and perception’ consists of the constructs ‘knowledge’ (on vitamin A and OFSP),’perceived susceptibility’ (perceptions on the susceptibility of vitamin A deficiency), ‘perceived severity’ (perceptions on the severity of vitamin A deficiency) and ‘health value’ (perceptions on the importance of health in general). These are followed by constructs around ‘beliefs and attitudes’, ‘health behavior identity’ (perception that it is healthy and good to eat OFSP), ‘attitudes toward behavior’ (feelings towards serving OFSP to their child) and ‘perceived barriers’ (perceived sensory (1) or agricultural (2)-related barriers that prevent the caretaker to serve OFSP to the child). The last category covers the external factors, ‘subjective norms’ (perceived social pressure on serving OFSP to their child), ‘control beliefs’ (perceived ability to make decisions in the household) and ‘cues to action’ (external triggers either (1) health-related or (2) activities that stimulate to serve OFSP to their child). *p<0.05, **p<0.01 (both two-tailed), Spearman’s correlation coefficients between constructs were calculated.

For the constructs (prior) behavior and intentional behavior a different scale was used, to reflect the frequency of (intended) consumption scored from 0–5: (0) never, (1) once a month, (2) 2–3 times a month, (3) once a week, (4) 2 or more times a week and (5) every day. The items of the majority of other constructs were scored from 1 to 5. For two constructs paired questions were asked; the construct ‘attitude toward behavior’ consisted of behavioral beliefs and the evaluation of these beliefs. For the construct ‘subjective norms’ these were the normative beliefs and motivation to comply. The scores of the beliefs were scored 1–5, whereas the evaluation of these beliefs and motivation to comply was scored with -2 to 2, after which the answers were multiplied resulting in a total score between -10 and 10. Total scores per construct were calculated for each caretaker, by adding all scores of the individual statements within a construct. The adjusted combined model of the TPB and the HBM (as we used for our study) can be found in Fig 1. In our case, compared to the original model presented by Sun et al.[18], the construct ‘prior behavior’ was a better predictor of behavior than the construct ‘behavioral intention’. Therefore, we swapped the constructs ‘behavior’ and ‘behavioral intention’ as shown in Fig 1. The questionnaire-based interview format is provided in the S1 Appendix.

#### Sweetpotato measurements

Dry matter content (%) was determined in triplicate for three randomly sampled raw roots of the four sweetpotato varieties. Dry matter content (%) was determined as the ratio of fresh weight compared to dry weight for the sweetpotato varieties. Sweetpotato samples were dried in an oven at 70 degrees °C until weight remained unchanged (on average 27 hours) [34]. Color charts were used to estimate beta-carotene levels for the different OFSP varieties [35].

#### Statistical analyses

The triangle and preference tests were analyzed using a binomial distribution. Critical values to determine the number of correct or agreeing choices were retrieved from statistical tables [27]. Sample sizes were sufficient to do analyses on location level. Data from the acceptance test for individual attributes were treated as ordinal data. Non-parametric tests were used to analyze if there were significant differences between location and varieties, the Mann Whitney U test for independent samples and the Wilcoxon Rank test for paired samples. Mean liking was defined as the average of all attributes assessed.

For the cultural acceptability survey, multiple item constructs were tested for reliability using the Cronbach-α and item total correlation. Items were removed from the analysis in case of a low item total correlation <0.3 or if the Cronbach alpha of the construct increased significantly upon removal of an item. In total 25 items were excluded. For each respondent, a total score per construct was calculated by adding all individual scores. Spearman’s correlation was used to calculate bivariate associations within constructs. Multiple linear regression modeling was performed to build the models. Models were adjusted for interviewer, education level, age and location (if applicable).

Statistical tests were 2-tailed and p-values <0.05 were considered statistically significant. All statistical analyses were performed using SPSS (version 23.0.0.2, ICM Corp. Released 2015) for Macintosh.

## Results

### Sensory evaluation

To investigate the sensory acceptability of the four different sweetpotato varieties, a total of 270 adults and 60 children participated in a range of different tests. The overall mean age of the adults was 31.9 (±10.7) years; slightly more women were included (63%). For the children, the mean age was 4.5 ±0.8 years. The percentage of participants that reported to grow OFSP was significantly different between areas, 36% in Lilongwe versus 72% in Mangochi. The consumed sweetpotatoes were mainly from own production (52%), the market (33%) or purchased from other farmers (26%). Participants reported to consume sweetpotato mostly as a breakfast dish (98%), and to a much lesser extent at lunch or dinner (respectively 14% and 13%). The most common prepared dishes were boiled OFSP (74%), boiled OFSP mixed with peanut flour (called ‘Futali’, 18%) and roasted OFSP (6%). As a benefit of sweetpotato, 59% of the participants reported health and nutrition related reasons, and 17% reported it as a source of income. The main reason for not consuming more sweetpotatoes was the availability (59%). In the triangle test, blinded participants in both areas were able to observe the difference between the orange and control sweetpotato samples, in total 49 out of 66 participants pointed out the odd sample, where 29 right answers were needed for a significant difference (p<0.05) (Table 1).

**Table 1:** Results for the triangle test with OFSP and the control yellow sweetpotato variety, per location and total results.

	Total	Lilongwe	Mangochi
Number of participants	66	36	30
Minimum number of correct judgments needed (α = 0.05)	29	18	15
Correct judgments	49*	28*	21*
Triangle u test: μ0 = 1/3	0.74	0.78	0.70

* significant (p<0.001, α = 0.05)

### Adult consumers display preference for the non-OFSP control variety over the OFSP variety

**Fig 2:**
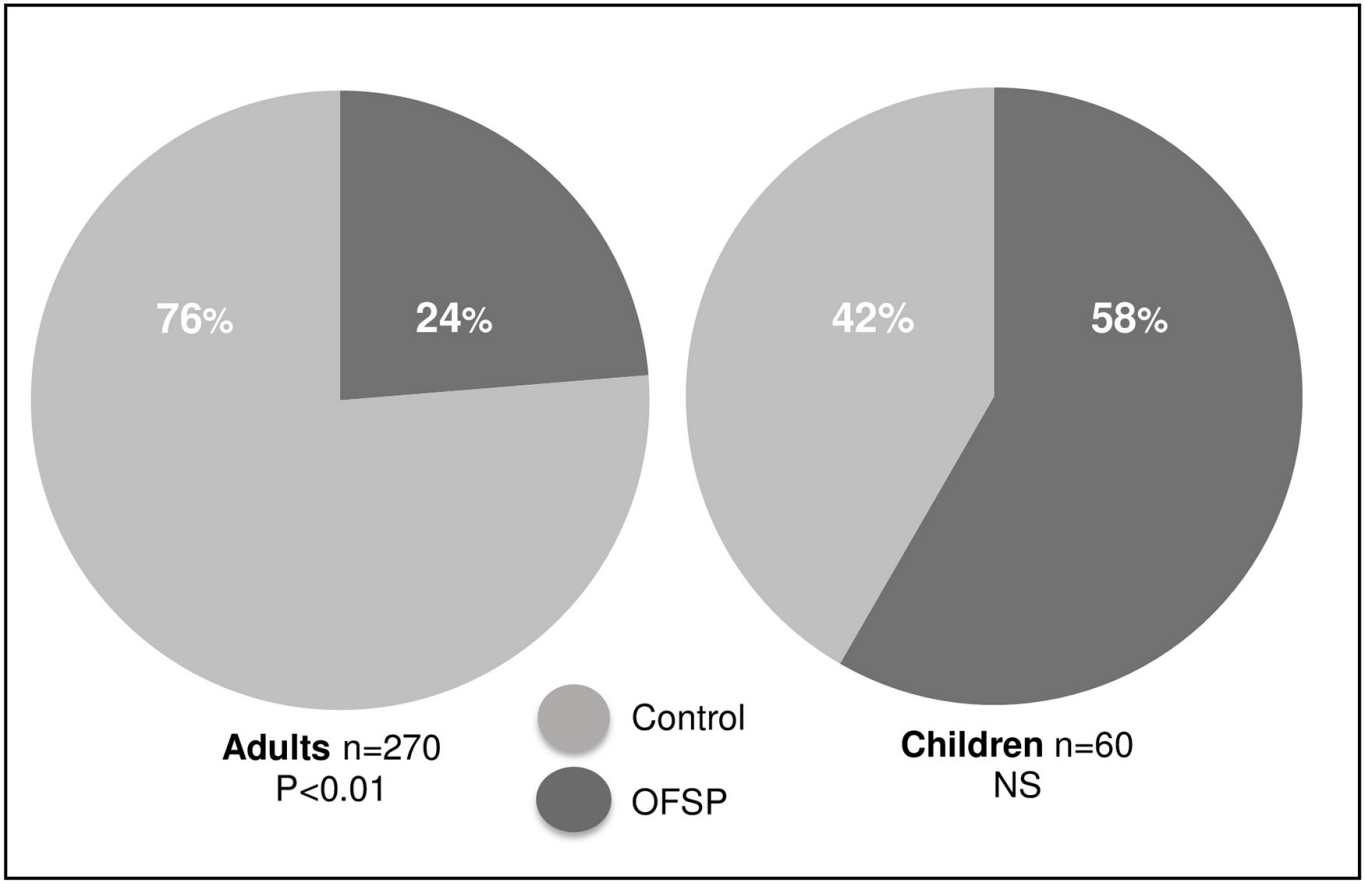
Results for the preference test with OFSP and a control sweetpotato variety. Adults significantly preferred the control variety (76.3%) over the orange variety, whereas more children preferred the OFSP (58.3%, not significant). * p<0.05.

For the preference test all participants (n = 270 adults, n = 60 children) were asked about their preference for either the yellow-fleshed control variety (Kenya) or the OFSP variety (Kadyaubwerere). Amongst the adults, sixty-four participants favored the OFSP variety (24%) (Fig 2 and S1 Table), due to sweetness (22%), odor (19%) and taste (19%). Two hundred and eleven participants favored the control variety (76%) because of sweetness (36%), starchiness (24%), and odor (13%). Color was also mentioned as a reason for preferring one of the varieties: 8% for the OFSP and 3% for the control sweetpotato variety. For the children (n = 60), the OFSP variety was preferred by 35 children, although this was not statistically significant (p>0.05) (Fig 2 and S1 Table). No significant differences in preference were found between locations for both adults and children.

To further investigate the difference in liking between varieties for seven different attributes, an acceptance test with four different varieties of sweetpotato was conducted (S2 Table). The color of the Zondeni and Kadyaubwere varieties were highest rated followed by the control variety Kenya; between these three no significant differences were found (p>0.05). Lowest rated was the Chipika variety, which was not significantly differently rated from the Kenya variety (p>0.05). For smell and texture only small differences were found in the liking of these attributes, for all varieties the median was 4. Hedonic scores of starchiness for the varieties Kenya and Zondeni (median 4) were significantly higher compared to Chipika and Kadyaubwerere (median 3) (p<0.05). Sweetness received a significantly different score for all varieties (p<0.05), Kenya was rated highest with a median of 5, followed by Zondeni (4), Kadyaubwerere (3) and Chipika (3). For the attribute taste, the median scores for Kenya were highest (5) and significantly higher than for the other three varieties (p<0.05). There was a significant difference in the scores of overall liking for all varieties (p<0.05), where Kenya was the preferred variety (5). No differences in liking for any of the seven attributes were found between locations for the Kenya variety. For the OFSP varieties Chipika and Kadyaubwere no difference was found for the overall liking between Lilongwe and Mangochi districts, all the other attributes were significantly differently evaluated. For the Zondeni variety a significant difference in hedonic scores between areas was found for all attributes, except for taste (p<0.05).

### Zondeni is the highest rated OFSP variety

After combining all attributes into a mean score for each variety (Table 2), a significant difference was found between all varieties. Overall, the control variety Kenya is liked most. In Mangochi however, the OFSP variety Zondeni is rated higher than Kenya, but this difference is not significant. Analysis of the difference in liking between the control and the OFSP varieties shows that there is on average a 0.50-point higher rating given for the control variety. There is a significant location effect between the areas, where the scores that were given for OFSP in Mangochi are much closer to the scores given for the control varieties (mean difference -0.22) than in Lilongwe (mean difference -0.76).

**Table 2:** Results for the acceptance test with calculated means of all attributes.

		Total	Lilongwe	Mangochi
		mean (SD)	mean (SD)	mean (SD)
**Mean of attributes**	Chipika (OFSP)	3.28 (0.98) a	3.08 (1.00) a	3.49 (0.91) a*
	Kadyaubwerere (OFSP)	3.51 (1.00) b	3.34 (0.99) b	3.69 (0.97) a*
	Zondeni (OFSP)	3.98 (0.96) c	3.60 (1.05) b	4.04 (0.80) b*
	Kenya (Control)	4.04 (0.81) d	4.13 (0.71) c	3.94 (0.90) b
	Overall liking OFSP	3.53 (0.69)	3.33 (0.71)	3.74 (0.62) *
**Mean differences**	Chipika- Control	-0.75 (1.24) a	-1.02 (1.31) a	-0.47 (1.10) a*
	Kadyaubwerere—Control	-0.52 (1.23) b	-0.77 (1.13) a	-0.27 (1.28) a*
	Zondeni—Control	-0.21 (1.30) c	-0.50 (1.38) b	0.09 (1.13) b*
	OFSP total—Control	-0.50 (1.04) b	-0.76 (1.06)	-0.22 (0.95)*

Means with a common letter in a column do not significantly differ at α = 0.05

* Significant difference between locations p<0.05

### Cultural acceptability study identifies opportunities and barriers for including OFSP in children’s diets by caretakers

To investigate the cultural acceptability of the OFSP varieties, a total of 302 caretakers were interviewed in a cultural acceptability study. The mean age of study participants was 31.9 (±9.1) years, almost all women (99.7% female). A household consisted on average of 5.9 persons (±1.8), where 74% of the caretakers had more than 3 children. In total 23.6% of the caretakers were illiterate, with most of them having attended only (part of) primary school (71%). The main household income source was from farming (56%). The majority of caretakers had consumed OFSP before (92.5%), with 20% reporting having grown OFSP in the last season and 60% reporting that they have grown any variety of sweetpotato in the last season. Additional descriptive information of the study population and difference between locations can be found in Table 3.

**Table 3:** Socio-demographic characteristics of caretakers in Lilongwe, Mangochi and total.

Mean age (SD)	Total	Lilongwe	Mangochi
	n = 302	n = 151	n = 151
	31.9 (9.1)	32.1 (8.1)	31.8 (10.1)
**Education (%)**			
No education	23.6	11.9	35.3
Primary school 1–4	35.9	35.8	36
Primary school 5–8	36.2	46.4	26
Secondary school	4.3	6	2.7
**Marital status (%)**			
Married	82.1	84.7	79.5
Separated	14.3	13.3	15.2
Widow	3.3	2.0	4.6
Single	0.3	-	0.7
**Number of children (%)**			
1–2 children	25.5	23.2	27.8
3–5 children	60.6	62.3	58.9
More than 5 children	13.2	13.2	13.2
**Income (%)**			
Farming	55.6	50.3	60.8
Casual labour	21.5	34.9	8.1
Remittances/gifts	7.4	-	14.9
Other	15.5	14.8	16.2

Sixty percent of caretakers reported that their children ate OFSP at least once a week when it was in season (between April and August), whereas 97% had the intention to feed their child OFSP once a week or more. The intention of feeding the children OFSP was much higher than current consumption when in season. The initial outcome construct of ‘behavioral intention’ showed very little variation in responses, which made it unsuitable to link the different constructs of the model to. Therefore, we used the construct ‘prior behavior’ instead as an outcome measure, which had a more even distribution of responses (and therefore was more suitable), which was included in the adjusted model in Fig 1.

Furthermore, over half of the caretakers agreed that OFSP is rich in vitamin A (59%), that vitamin A could improve eyesight (62%), and could prevent diseases (60%). Most caretakers agreed that children in the age between 2–5 years old are at risk of developing vitamin A deficiency (69%) and almost the same proportion of caretakers also considered their own child being at risk of developing vitamin A deficiency (65%). Most caretakers agreed with the statements that ‘vitamin A deficiency makes the child more frequently ill’ (77%) and ‘lack of vitamin A can lead to stunted growth of my child’ (71%). Caretakers acknowledged that it was very important to them that their child can see properly during dusk (96%) and has a good health (99%). The majority of the caretakers (88%) were convinced that eating OFSP was good for their child, while the remaining 12% gave a neutral response to this question.

The majority of participants agreed that OFSP has an attractive color (86%) and that it tastes well (96%). Over one third of respondents indicated that they would rather sell OFSP than consume it themselves (37%). Caretakers indicated that provision of OFSP vines would make them decide to cultivate and prepare OFSP for their child (98%) and that information sessions on the benefits of OFSP would convince them to feed OFSP to their children (94%). Most caretakers agreed that other cues to prepare OFSP for their child were: (a) if their child was sick, (b) would have vitamin A deficiency or (c) would have problems with seeing properly during dusk or dawn (65–71%). The most influencing opinions on food preparation for caretakers were the opinions from health workers (96%), the child growth centers (90%) and health extension workers (80%). The opinions of friends and neighbors are much less valued in making decisions on what food to prepare for their children (both 53%). Furthermore, the majority of caretakers indicated that they would regret it if they would not give OFSP to their child (91%). Table 4 provides an overview of the different constructs. Cronbach-α scores ranged from 0.53 to 0.81, which demonstrated a medium reliability for most of the constructs, median scores of the constructs were ranging from 3 to 51.

**Table 4:** Internal consistency, median scores, range of score and item examples of all constructs (n = 302).

Constructs	Items	Cronbach	Median	Range	Item example
Knowledge	4	0.69	16	8–20	OFSP is rich in vitamin A
Perceived susceptibility	2	0.58	8	2–10	My child is at risk of developing VAD
Perceived severity	8	0.81	33	10–40	Lack of VA can make my child malnourished
Health value	4	0.64	20	12–20	The health of my child is the most important thing in my life
Health behavior identity	1	-	5	1–5	Eating OFSP is good for my child
Perceived barriers-1	3	0.75	4	3–15	I worry about the orange colour of orange-fleshed sweetpotato
Perceived barriers-2	8	0.63	22	9–30	I would rather sell OFSP than keep it for consumption
Attitude	7	0.53	51	11–70	OFSP tastes well/ I find it important that my child eat foods that tastes well
Control beliefs	3	0.74	15	3–15	Other people decide what food I buy for my household
Subjective norm	8	0.81	22	-16-80	My parents advise me to prepare sweetpotato/The opinion of my parents about food is important to me
Cues to action-1	3	0.69	12	3–15	When my child is sick, I will prepare OFSP
Cues to action-2	7	0.76	32	7–35	Information sessions about the benefits of orange-fleshed sweetpotato Potato would encourage me to prepare OFSP for my child
Behavioral intention	1	-	5	1–5	How often do you think you will prepare orange fleshed sweetpotato for your child in the future if it is available?
Anticipated affect	3	0.57	11	3–15	If I don’t give OFSP to my child I will regret it
Prior behavior	1	-	3	1–5	How often did your child eat orange-fleshed sweetpotato when it was in season?

Fig 1 shows the correlations between the different constructs of the model. Within the background and perception section significant correlations were found between health behavior identity and the constructs ‘knowledge’(r = 0.230), ‘perceived susceptibility’ (r = 0.168) and ‘perceived severity’(r = 0.219, all p<0.01). For the beliefs and attitudes section ‘health behavior identity’ was correlated with the construct ‘attitude toward behavior’ (r = 0.159, p<0.01) and ‘perceived barriers -1’ (r = -0.118, p<0.05). Perceived barriers-1 are barriers related to color, taste and starch content of the OFSP. Only the construct ‘attitude toward behavior’ was significantly correlated to the construct ‘prior behavior’ (r = 0.149 p<0.05). For the external factors, the constructs ‘subjective norms’(r = 0.158) and cues to action-2’ (r = 0.187) were significantly correlated with prior behavior (p<0.01). Cues to action-2 are related to activities and recommendations by others promoting OFSP. The construct ‘anticipated affect’ was significantly correlated with ‘behavioral intention’ (r = 0.145, p<0.05), suggesting that a higher regret of not giving OFSP to the child leads to higher behavioral intention to give OFSP to the child. However, the construct ‘anticipated affect’ is not correlated with prior behavior (r = -0.012). No significant correlation was found between the constructs ‘behavior’ and ‘behavioral intention’.

### Attitudes towards behavior and subjective norms can predict prior behavior in relation to caretakers serving their child OFSP

To further assess the relationship between multiple constructs and to investigate which constructs can predict prior behavior, multiple linear regression was used. An overview of the relative contribution of the constructs to prior behavior, using three different models is provided in Table 5. Overall, the constructs could explain a small percentage of the total variance in predicting the behavior. Model 1, ‘background and perception’, explained 10% of the variance in ‘health behavior identity’. No significant predictors were found in the total model, which was also the case when analyzed on location level. Model 2 explained 24% of the variance of the internal factors influencing the prior behavior of the caretakers to give OFSP to their children. Only ‘attitudes towards behavior’ (β = 0.14) was a significant predictor (p = 0.01) of prior behavior. When the model was predicted only for Lilongwe, ‘health behavior identity’ (β = 0.24) was a significant predictor of prior behavior (p = 0.01). For Mangochi ‘attitude toward behavior’ (β = 0.22) was a significant predictor (p = 0.01) of prior behavior. For model 3, predicting prior behavior using external factors, the construct ‘subjective norms’ was a significant predictor (β = 0.25, p = 0.00), also when the model was run for either of the two research locations.

**Table 5:** Overview of all models: Predictors for health behavior identity (model 1) and prior behavior of consuming OFSP (model 2 and 3) among children of caretakers in Malawi (n = 302).

	Total			Lilongwe			Mangochi		
	Standardized β	p	Adjusted R square	Standardized β	p	Adjusted R square	Standardized β	p	Adjusted R square
**Model 1**									
Dependent variable: Health behavior identity			0.10			0.27			0.05
Knowledge	0.06	0.37		0.07	0.45		0.07	0.48	
Perceived susceptibility	0.02	0.77		0.12	0.22		-0.14	0.16	
Perceived severity	0.10	0.15		0.05	0.63		0.18	0.07	
Health value	0.03	0.64		0.05	0.48		-0.03	0.70	
**Model 2**									
Dependent variable: Prior behavior			0.24			0.12			0.41
Health behavior identity	0.09	0.13		0.24	0.01*		-0.06	0.39	
Perceived barriers -1	0.07	0.25		0.08	0.39		0.08	0.30	
Perceived barriers -2	0.04	0.55		0.04	0.65		0.03	0.71	
Attitude toward behavior	0.14	0.03*		0.05	0.67		0.19	0.03*	
Anticipated affect	0.03	0.58		0.06	0.49		0.00	0.99	
**Model 3**									
Dependent variable: Prior behavior			0.25			0.13			0.38
External control beliefs	0.13	0.08		0.17	0.16		0.07	0.36	
Subjective norms	0.23	0.00*		0.26	0.02*		0.21	0.01*	
Cues to action-1	0.02	0.81		0.00	0.99		-0.03	0.75	
Cues to action-2	-0.08	0.22		-0.14	0.16		-0.09	0.32	

Adjusted for interviewer, age, level of education, location (only for total model)

* Significant predictor in the model (p<0.05)

## Discussion

For biofortified crops to have an impact on micronutrient intake, it is required that biofortified varieties are consumed in sufficient quantities by the vulnerable target populations, in particular to improve maternal and child health. In addition to dissemination barriers (e.g., ineffective seed systems) that can limit access by smallholder farmers to healthy planting materials (seeds, vines) of new biofortified varieties [36], additional barriers to acceptability and sustained consumption can arise due to biofortified varieties not having equivalent or improved sensory characteristics, or due to a lack of preference for the varieties by the caretakers of children.

The first objective of our study was to assess sensory acceptability of the OFSP varieties in comparison with a control variety. The difference in preference for the varieties per location highlights the importance of conducting sensory evaluation research in different areas, to be able to adjust variety dissemination initiatives to local preferences where possible, particularly to increase acceptability of varieties.

In our study, caretakers in Lilongwe significantly preferred the yellow-fleshed variety over the OFSP varieties, whereas children did not significantly prefer either. In contrast, research amongst children and mothers in Tanzania found that higher mean acceptability scores were observed for OFSP in comparison with pale–fleshed sweetpotato varieties although children gave significantly lower scores than mothers [29]. These contrasting results could potentially be due to regional differences in acceptability, and/or differences in % dry matter, color, flavor, smell and other important sensory characteristics. The preference for the control variety expressed in our study group may be of concern when promoting OFSP in Malawi, since foods liked by mothers are more likely to be offered to their children [37]. This would decrease the exposure of OFSP to children in our study population as the mothers are the primary caregivers. To address this potential barrier, it would be important to have an effective strategy to promote OFSP amongst mothers, which makes it more likely they will feed it to their children.

Dry matter has been identified as an important varietal trait when comparing OFSP to other pale-fleshed sweetpotato varieties, and has been reported as an important attribute for the liking of OFSP by consumers [38, 39]. The OFSP varieties used for the triangle test differed in their dry matter content, which has been shown to make it easier to identify the odd sample [27]. However, our aim in this study was to compare the most promising OFSP variety (based on yield and beta-carotene content), Kadyaubwerere, with the control variety used by farmers and in households. According to a study in Kenya, children have a preference for OFSP varieties with lower dry matter content, whereas adults prefer high dry matter content (>27%) [40], which was not recapitulated in our study. Analysis of the dry matter content of the OFSP varieties used in our study demonstrated that the control variety Kenya had the highest dry matter content (39.2%), followed by Chipika and Zondeni (respectively 34.6% and 34.3%), with the lowest dry matter content found for Kadyaubwerere (29.8%). In our study, the dry matter content factor alone cannot explain the difference in liking between Chipika (lowest rated OFSP) and Zondeni (highest rated OFSP). Other studies on sensory characteristics of OFSP and cream fleshed sweetpotato varieties have concluded that major varietal differences are differences in color, dry mass, sweet flavor and maltose content [38]. These characteristics could likely also explain the differences in liking of the OFSP varieties in our tests. Therefore, further research is needed to test the relationship between the hedonic test results with sensory characteristics of the different varieties to be able to explain the differences in liking in more detail. It is also important to take into account that textural traits can potentially be influenced by genotype-environment interactions, which can complicate the testing and selection of varieties for consumer acceptance and breeding for improved textural traits [39].

Another angle that provides opportunities for increasing the sensory acceptability of OFSP is researching the effect of information provision. Research showed that nutrition information combined with tasting OFSP is positively weighted and integrated by the consumer to form emotions, that can be associated with product acceptance [41]. A review summarizing acceptance studies on biofortified crops concluded that information on the health benefits is an important determinant of acceptance [42].

Our acceptance tests revealed high scores (means are >3) for all sweetpotato varieties, which indicates that all of the sweetpotato varieties are accepted. Since uptake of OFSP among a population who’s source of income is mainly farming not only depends on sensory acceptability but also on production and farming system attributes (e.g. yield, resistance to pests and diseases) [43, 44], such additional factors determining adoption for cultivation and marketing should also be taken into account (see S3 Table). While the sensory acceptability for the Zondeni variety was high, the potential yields for Chipika and Kadyaubwerere are 35 t / ha under ideal circumstances, whereas Zondeni’s yield potential is only 8–16 t / ha. Therefore, from a food security point of view based on aggregate supply of sweetpotato the promotion of the Zondeni variety amongst smallholders might not be justified. On the other hand, from a nutritional perspective, the beta-carotene levels of the different OFSP varieties are also an important factor to take into account. The Chipika variety has a much lower beta-carotene content (3500 μg/100g) compared to the other OFSP varieties Kadyaubwerere and Zondeni (respectively 8900 and 9000 μg/100g) and the control variety (770 μg/100g) (see S3 Table for more data on characteristics of the different varieties used). We acknowledge that measurement of beta-carotene using the HPLC method would be favourable [42], since estimations made with color charts are less precise. However, even with our estimation approach, the differences in potential yield and beta-carotene content of the different varieties (resulting in different nutritional yields) are clear.

It is possible that the preference for the Zondeni variety might be due to an inertia effect as it was the first OFSP variety that was introduced, 3 years before the other two OFSP varieties that were tested were introduced, so participants had more exposure to this variety. It is also possible that the much higher yield of the other OFSP varieties compared to Zondeni might act as a driver for smallholder farmer adoption, that is sufficient to override the relatively small differences in liking between the varieties.

The second objective of our study was to take a cultural acceptability approach to identify the constructs that contribute most to behavior of caretakers serving their children OFSP. Our findings indicate that this behavior was strongest correlated with the constructs ‘subjective norms’ and ‘attitudes toward behavior’. The discrepancy between the intention and prior behavior shows the caretakers’ difficulty to implement the behavior possibly through various personal and environmental control factors [45, 46]. Depending on the type of behavior, the strength of the intention-behavior relationship can vary widely, and the discrepancy is larger when multiple steps have to be taken before the intention can be realized into the behavior [45].

Using prior behavior as an outcome measure, the constructs could only explain a small portion of the total variance for predicting the caretaker’s frequency of serving OFSP to their children (23–29%). This is in concordance with other studies [18, 21–23], that found similar low explained variances by using the intention as an outcome measure. Therefore, an addition of the construct anticipated affect was made to the model, which explained an additional 3% of total variance. Despite its small contribution, the anticipated affect is important to take into account [33]. However, our study reveals that there remain other unknown factors that will be necessary to identify to explain the remaining variance.

Most of the respondents tended to agree with the statements in the cultural acceptability survey, and because of that, scores were high compared to the ranges possible. This high level of agreement to the statements can be due to different reasons. Firstly, it might be related to unfamiliarity to the behavior. We attempted to prevent the behavior unfamiliarity effect by only enrolling people in the survey that knew OFSP and selecting areas where it was introduced by ongoing agri-development programs and by including both negative and positively phrased questions. The intention to consume OFSP was very high, but at the same time the access to the OFSP was low, since the roots were not widely available in markets and the planting material was hard to access. Unfamiliarity with the behavior makes it less likely that there are strong beliefs towards the statements, and therefore the respondents might have had difficulty to decide on their level of agreement or disagreement. Secondly, another important factor that might have influenced our findings is that in general, respondents attempt to understand the goal of the research, in order to tailor their results which they hope will benefit themselves, their family or their community in future [47]. By giving positive responses respondents might have hoped that the survey showed their community to be a good place to continue the programming on OFSP, to be able to receive planting material or more support.

Concerning the constructs within the section background and perception it was found that the construct ‘health behavior identity’ was significantly correlated with the internal factors ‘knowledge’, ‘perceived susceptibility’ and ‘perceived severity’. However, none of these constructs were predictors in the model. In our study, we can conclude that specific knowledge on vitamin A and the threats of vitamin A deficiency are more likely to positively influence caretakers’ behavior to serve OFSP to their children than a more general knowledge on health, which is reflected by the construct ‘health value’.

The prior behavior of caretakers serving their child OFSP was predicted (p<0.05) by the constructs ‘attitude towards behavior’ and ‘subjective norms’. The construct ‘attitudes toward behavior’ was a good predictor within the section beliefs and attitudes, which confirms results of other studies [22, 48]. These attitudes were determined by questions about beliefs on serving the child OFSP and the importance of these beliefs for the caretakers. The most important attitudes were the (sweet) taste, the attractive appearance, that it can cure and protect against diseases, and that it is easy to prepare.

The construct ‘subjective norms’ was a good predictor within the external factors. It reflects social pressure, which can be explained as the influence other people have on the decision whether a caretaker will serve OFSP to their children or not. In particular, the opinions of the extension workers, health workers and parents were highly valued, according to the responses. It has been highlighted that Malawi is a collectivist society [49], which can mean individuals put the priority of the group above the priorities of the individual [50]. In addition, the values of extended family and the community have a major influence on the behavior of the individual. This is important to take into account when promoting OFSP, to not only focus on positive attitudes and knowledge of women, but to also include a wider range of social ‘influencers’. Other studies have found that the subjective norms were correlated. However, they were not a good predictor of the intention [22, 51] or did not find any correlation [18, 21].

The perceived economic and health benefits of OFSP in Malawi have been studied among farmers of OFSP [52]. The health benefits that were most frequently mentioned were increased energy, improved eyesight and the perception that OFSP is good for healthy bodies. From an economic perspective benefits cited were the ability to invest the income retrieved by selling OFSP (vines) in housing, livestock and food. In addition, women mentioned an increased self-esteem through the increased incomes. The most important benefits of producing and consuming OFSP can be used in information sessions and nutrition sessions where knowledge on the OFSP is communicated to potential consumers and/or farmers. These benefits would also help to create positive attitudes towards the behavior of consuming OFSP and increasing the already high acceptability of OFSP.

### Conclusions

Overall, our study reveals that biofortified OFSP varieties are well accepted in Lilongwe and Mangochi districts in Malawi from both a cultural and sensory perspective. However, we find that there is a preference for the yellow-fleshed control variety and the Zondeni variety which is high in vitamin A. Our cultural acceptability analysis indicates that attitudes toward behavior and subjective norms were correlated to, and important predictors of the caretakers’ behavior of serving their child OFSP. Our study findings provide guidance and direction for improvement of ongoing and planned programs for increasing the uptake of OFSP in Malawi among households with children. We consider that there is a need to conduct a follow-on in-depth study quantifying sensory characteristics (sweetness, maltose concentration, dry matter) of the OFSP varieties and a more accurate quantification of the beta-carotene levels of the varieties to be able to unravel favourable traits by linking this information to the hedonic test results. Our results also indicate that there is both a need and an opportunity to promote a more diversified use of OFSP, as it is currently almost exclusively consumed as a breakfast snack (where the OFSP are mostly prepared through boiling), or in a dish called Futali. The high energy density of OFSP should be taken into account, to make sure it is a good and nutritious replacement when increasing intake or diversifying the use. The ongoing programs for promotion of uptake of OFSP varieties in Malawi will need to decide which specific OFSP varieties to promote based on criteria that include sensory acceptability, beta-carotene content or agricultural characteristics as well. For increasing adoption and consumption of OFSP to improve maternal and child health in Malawi, there is an additional opportunity to focus on positive attitudes and identify and include important influencers around the caretaker in the promotion strategy to increase the frequency of caretakers serving OFSP to their children.

Overall, while biofortified crops such as OFSP have major promise for combatting hidden hunger micronutrient deficiencies, our study highlights that consideration of sensory and cultural attributes that can influence both acceptability and consumption amongst smallholder farmers and households can improve impact pathways for biofortified crops.

## Supporting information

S1 Table Results for the preference test with OFSP and a control yellow-fleshed sweetpotato variety among adults (n = 270) and children (n = 60) per location. (TIF)

S2 Table Results for the acceptance test with 3 OFSP varieties and a control variety, per area and total. (TIF)

S3 Table Beta-carotene content and dry matter (%) content for the various sweetpotato varieties. (TIF)

S1 Appendix Questionnaire format for sensory and cultural acceptability questionnaire. (XLSX)

## References

[1] Bouwkamp JC. Sweet potato products: a natural resource for the tropics. 1st ed Boca Raton CRC Press, Inc.; 1985.

[2] Low JW, Mwanga RO, Andrade M, Carey E, Ball A-M. Tackling vitamin A deficiency with biofortified sweetpotato in sub-Saharan Africa. Global Food Security 2017. 10.1016/j.gfs.2017.01.004 PMC5614018 28989861

[3] Bovell‐Benjamin AC. Sweet potato: a review of its past, present, and future role in human nutrition. Advances in food and nutrition research. 2007;52:1–59. 10.1016/S1043-4526(06)52001-7 17425943

[4] van Jaarsveld PJ, Faber M, Tanumihardjo SA, Nestel P, Lombard CJ, Benadé AJS. β-Carotene–rich orange-fleshed sweet potato improves the vitamin A status of primary school children assessed with the modified-relative-dose-response test. The American journal of clinical nutrition. 2005;81(5):1080–7. 10.1093/ajcn/81.5.1080 15883432

[5] (WHO) WHO. Global prevalence of vitamin A deficiency in populations at risk 1995–2005 Geneva: World Health Organization, 2009.

[6] Department of Nutrition HaAitOoPaCD-O, Ministry of Health (MOH), National Statistics Office (NSO), UNICEF and CDC. The national micronutrient survey 2009. Lilongwe, Malawi 2011.

[7] National Statistical Office (NSO) CHSUCM, Centers for Disease Control and Prevention (CDC), and Emory University. Malawi Micronutrient Survey 2015–16: Key Indicators Report. Atlanta, GA, USA: NSO, CHSU, CDC and Emory University 2016.

[8] WHO. Serum retinol concentrations for determining the prevalence of vitamin A deficiency in populations. In: Organization WH, editor. Geneva: Vitamin and Mineral Nutrition Information System; 2011.

[9] ICF NSONMa. Malawi Demographic and Health Survey 2015–16. Zomba, Malawi, and Rockville, Maryland, USA: NSO and ICF, 2017.

[10] Bouis HE, Saltzman A. Improving nutrition through biofortification: A review of evidence from HarvestPlus, 2003 through 2016. Global Food Security. 2017;12:49–58. 10.1016/j.gfs.2017.01.009 28580239 PMC5439484

[11] Olson JA. Provitamin A function of carotenoids: the conversion of beta-carotene into vitamin A. The Journal of nutrition. 1989;119(1):105–8. 10.1093/jn/119.1.105 2643691

[12] Hotz C, Loechl C, de Brauw A, Eozenou P, Gilligan D, Moursi M, et al A large-scale intervention to introduce orange sweet potato in rural Mozambique increases vitamin A intakes among children and women. British Journal of Nutrition. 2012;108(01):163–76. 22018075 10.1017/S0007114511005174

[13] CIP Sr. Orange Fleshed Sweetpotato Varieties for Malawi / Mitunda ya mbatata yofiira mkat yaku Malawi In: (CIP) IPC, editor. 2015. p. 12.

[14] Tomlins K, Owori C, Bechoff A, Menya G, Westby A. Relationship among the carotenoid content, dry matter content and sensory attributes of sweet potato. Food Chemistry. 2012;131(1):14–21.

[15] TAKAHATA Y, NODA T, NAGATA T. HPLC determination of β-carotene content of sweet potato cultivars and its relationship with color values. Japanese Journal of Breeding. 1993;43(3):421–7.

[16] Birol E, Meenakshi J, Oparinde A, Perez S, Tomlins K. Developing country consumers’ acceptance of biofortified foods: a synthesis. Food Security. 2015;7(3):555–68.

[17] Talsma EF, Melse-Boonstra A, Brouwer ID. Acceptance and adoption of biofortified crops in low-and middle-income countries. Nutr Rev. 2017;75(10):798–829. 10.1093/nutrit/nux037 29028269 PMC5914320

[18] Sun X, Guo Y, Wang S, Sun J. Predicting iron-fortified soy sauce consumption intention: application of the theory of planned behavior and health belief model. Journal of Nutrition Education and Behavior. 2006;38(5):276–85. 10.1016/j.jneb.2006.04.144 16966048

[19] Ajzen I. The theory of planned behavior. Organizational behavior and human decision processes. 1991;50(2):179–211.

[20] Janz NK, Becker MH. The health belief model: A decade later. Health education quarterly. 1984;11(1):1–47. 10.1177/109019818401100101 6392204

[21] Talsma EF, Melse-Boonstra A, de Kok BP, Mbera GN, Mwangi AM, Brouwer ID. Biofortified cassava with pro-vitamin A is sensory and culturally acceptable for consumption by primary school children in Kenya. PloS one. 2013;8(8):e73433 10.1371/journal.pone.0073433 24023681 PMC3758265

[22] Fanou-Fogny N, van Dam B, Koreissi Y, Dossa RA, Brouwer ID. Factors predicting consumption of fonio grain (Digitaria exilis) among urban Malian women of reproductive age. Journal of nutrition education and behavior. 2011;43(4):219–28. 10.1016/j.jneb.2010.03.008 21377935

[23] Macharia-Mutie CW, Van de Wiel AM, Moreno-Londono AM, Mwangi AM, Brouwer ID. Sensory acceptability and factors predicting the consumption of grain amaranth in Kenya. Ecology of food and nutrition. 2011;50(5):375–92. 10.1080/03670244.2011.604584 21895418

[24] Services DoCCaM. Climate of Malawi 2006 [20–1–2018]. https://www.metmalawi.com/climate/climate.php.

[25] National Statistics Office. Integrated household survey 2016–2017. Malawi: 2017.

[26] Aberman N-L, Meerman J, Benson T. Mapping the linkages between agriculture, food security and nutrition in Malawi: Intl Food Policy Res Inst; 2015.

[27] Lawless HT, Heymann H. Sensory evaluation of food: principles and practices: Springer Science & Business Media; 2010.

[28] International Potato Center (CIP) SSA region. Orange Fleshed Sweetpotato Varieties for Malawi / Mitunda ya mbatata yofiira mkati yaku Malawi 2015.

[29] Tomlins K, Ndunguru G, Stambul K, Joshua N, Ngendello T, Rwiza E, et al Sensory evaluation and consumer acceptability of pale‐fleshed and orange‐fleshed sweetpotato by school children and mothers with preschool children. Journal of the Science of Food and Agriculture. 2007;87(13):2436–46.

[30] Kemp S, Hollowood T, Hort J. Sensory evaluation: a practical handbook. 1st ed Chichester: Wiley Blackwell; 2009.

[31] Guinard J-X. Sensory and consumer testing with children. Trends in Food Science & Technology. 2000;11(8):273–83.

[32] Den Hartog AP, Van Staveren WA, Brouwer ID. Food habits and consumption in developing countries—manual for field studies. 1st ed Wageningen: Wageningen Academic Publishers; 2006.

[33] Ajzen I, Sheikh S. Action versus inaction: anticipated affect in the theory of planned behavior. Journal of Applied Social Psychology. 2013;43(1):155–62.

[34] AOAC. Official methods of analysis (18th edn, rev. 2). Gaithersburg, Marlyland, USA: AOAC International; 2007.

[35] Burgos B, Carpio R, Sanchez C, Paola S, Eduardo P, Espinoza J, et al., editors. A color chart to screen for high β-carotene in OFSP breeding. 15th Triennial Symposium of the International Society for Tropical Root Crops, Lima, Peru; 2009.

[36] McGuire S, Sperling L. Seed systems smallholder farmers use. Food Security. 2016;8(1):179–95.

[37] Skinner JD, Carruth BR, Bounds W, Ziegler PJ. Children’s food preferences: a longitudinal analysis. Journal of the American Dietetic Association. 2002;102(11):1638–47. 12449287 10.1016/s0002-8223(02)90349-4

[38] Laurie SM, Faber M, Calitz FJ, Moelich EI, Muller N, Labuschagne MT. The use of sensory attributes, sugar content, instrumental data and consumer acceptability in selection of sweet potato varieties. Journal of the Science of Food and Agriculture. 2013;93(7):1610–9. 10.1002/jsfa.5932 23132727

[39] Anderson L, Gugerty MK. Root, Tuber, and Banana Textural Traits A Review of the Available Food Science and Consumer Preferences Literature. 2015.

[40] Hagenimana V, Low J. Potential of orange-fleshed sweet potatoes for raising vitamin A intake in Africa. Food and Nutrition Bulletin. 2000;21(4):414–8.

[41] Lagerkvist CJ, Okello J, Muoki P, Heck S, Prain G. Nutrition promotion messages: The effect of information on consumer sensory expectations, experiences and emotions of vitamin A-biofortified sweet potato. Food quality and preference. 2016;52:143–52.

[42] Talsma EF, Melse-Boonstra A, Brouwer ID. Acceptance and adoption of biofortified crops in low-and middle-income countries: a systematic review. Nutrition reviews. 2017;75(10):798–829. 10.1093/nutrit/nux037 29028269 PMC5914320

[43] Berre D, Corbeels M, Rusinamhodzi L, Mutenje M, Thierfelder C, Lopez-Ridaura S. Thinking beyond agronomic yield gap: Smallholder farm efficiency under contrasted livelihood strategies in Malawi. Field Crops Research. 2017;214:113–22.

[44] Tittonell P, Giller KE. When yield gaps are poverty traps: The paradigm of ecological intensification in African smallholder agriculture. Field Crops Research. 2013;143:76–90.

[45] Randall DM, Wolff JA. The time interval in the intention‐behaviour relationship: Meta‐analysis. British Journal of Social Psychology. 1994;33(4):405–18.

[46] Glanz K, Rimer BK, Viswanath K. Health behavior and health education: theory, research, and practice: John Wiley & Sons; 2008.

[47] Miller K, Msiyaphazi Zulu E, Cotts Watkins S. Husband—Wife Survey Responses in Malawi. Studies in Family Planning. 2001;32(2):161–74. 11449864 10.1111/j.1728-4465.2001.00161.x

[48] Nejad LM, Wertheim EH, Greenwood K. Comparison of the health belief model and the theory of planned behavior in the prediction of dieting and fasting behavior. Sensoria: A Journal of Mind, Brain & Culture. 2005;1(1):63–74.

[49] Sulamoyo DS. Creating Opportunities for Change and Organization Development in Southern Africa. 1st ed Charlotte, NC: Information Age Publishing; 2012.

[50] Kâğitçibaşi Ç. A critical appraisal of individualism and collectivism: Toward a new formulation. Thousand Oaks, CA: Sage; 1994 52–65 p.

[51] Rah JH, Hasler CM, Painter JE, Chapman-Novakofski KM. Applying the theory of planned behavior to women’s behavioral attitudes on and consumption of soy products. Journal of nutrition education and behavior. 2004;36(5):238–44. 15707546 10.1016/s1499-4046(06)60386-2

[52] Mudege NN, Mayanja S, Muzhingi T. Women and men farmer perceptions of economic and health benefits of orange fleshed sweet potato (OFSP) in Phalombe and Chikwawa districts in Malawi. Food Security. 2017;9(2):387–400.

