# Supplementary figures and images for: Sensory and cultural acceptability tradeoffs with nutritional content of biofortified orange-fleshed sweetpotato varieties among households with children in Malawi
Source: PLoS One. 2018 Oct 18;13(10):e0204754. doi: 10.1371/journal.pone.0204754 (PMC6193634; doi:10.1371/journal.pone.0204754)

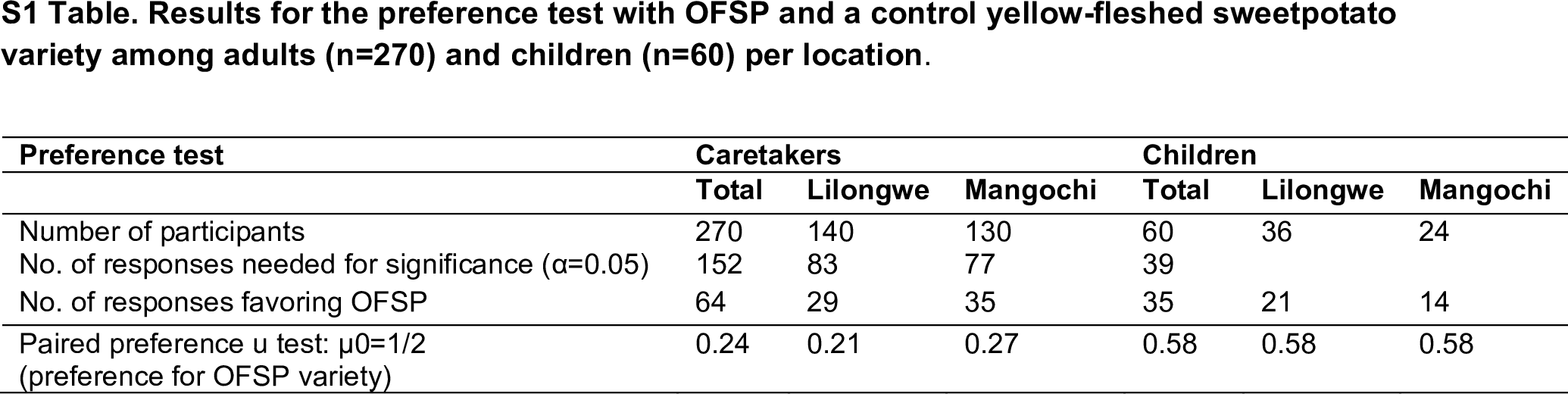

Supplement: S1 Table — (TIF) [file pone.0204754.s001.tif]

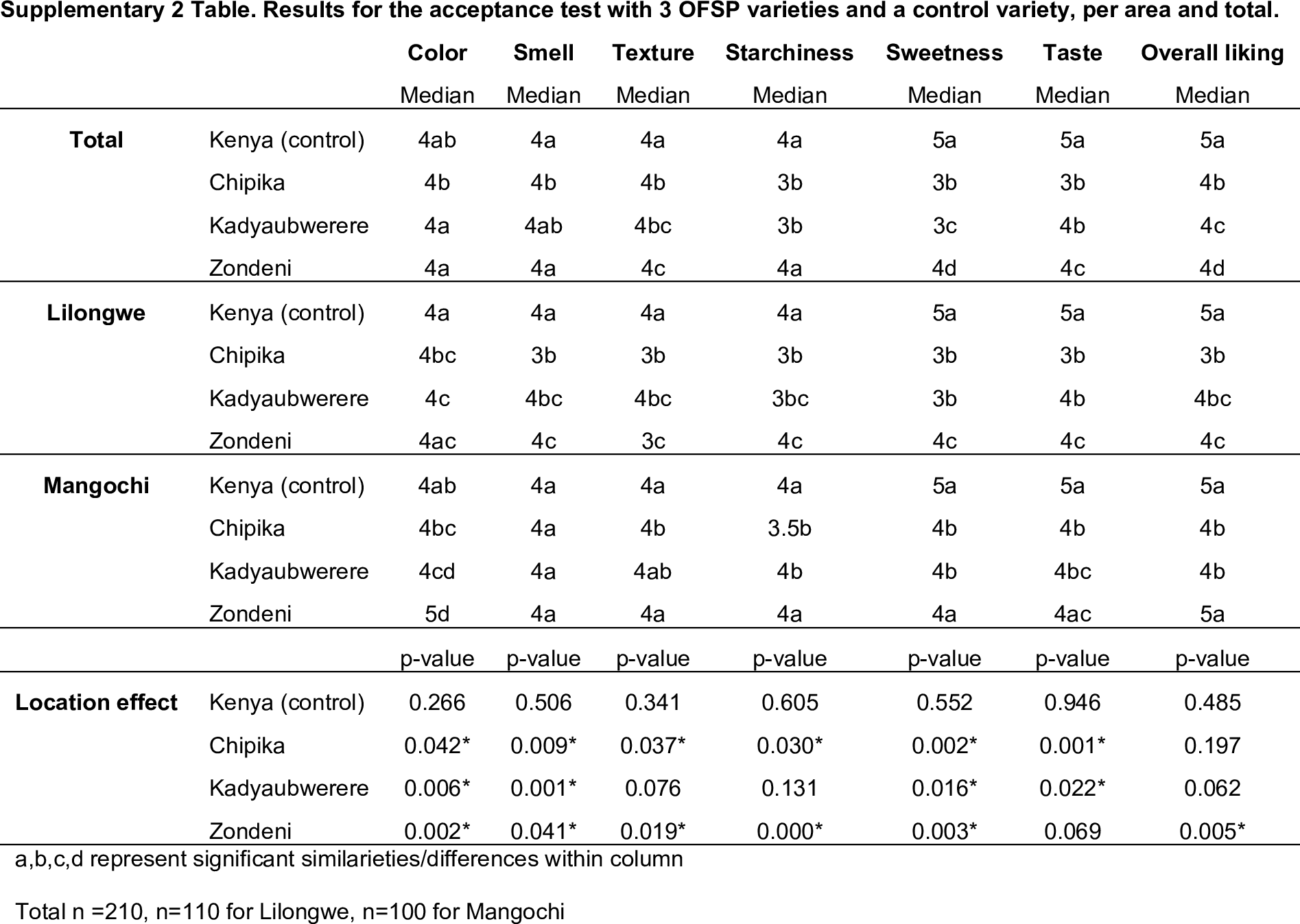

Supplement: S2 Table — (TIF) [file pone.0204754.s002.tif]

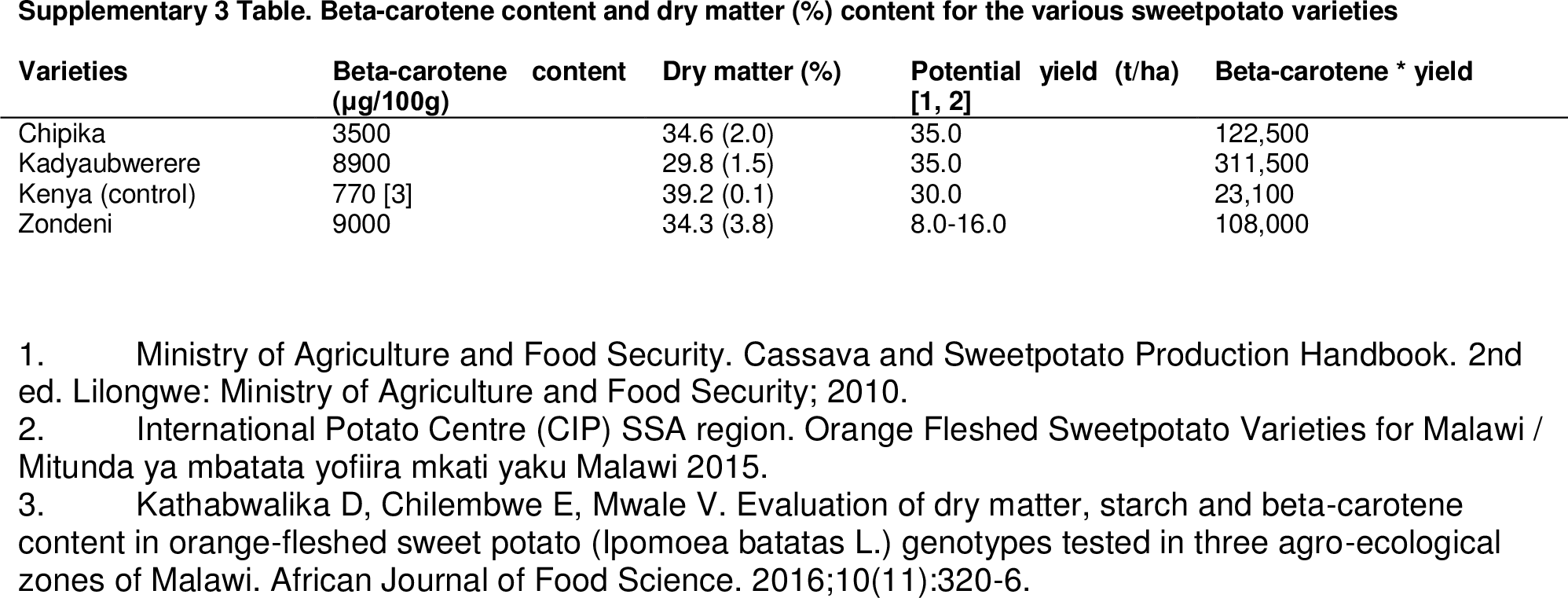

Supplement: S3 Table — (TIF) [file pone.0204754.s003.tif]
